# Supplementary material for: Dynamic relational event modeling: Testing, exploring, and applying
Source: PLoS One. 2022 Aug 1;17(8):e0272309. doi: 10.1371/journal.pone.0272309 (PMC9342787; doi:10.1371/journal.pone.0272309)
Supplement: S1 File — (PDF) [file pone.0272309.s001.pdf]

Supporting figures for

Dynamic relational event modeling: Testing, exploring and applying

Marlyne Meijerink-Bosman, Roger Leenders and Joris Mulder

February 22, 2022

List of Figures

1 Baseline - Results 2
2 Baseline - Bias 3
3 Baseline - Average estimated SE 4
4 Z of sender- Results 5
5 Z of sender - Bias 6
6 Z of sender - Average estimated SE 7
7 Difference in Z - Results 8
8 Difference in Z - Bias 9
9 Difference in Z - Average estimated SE 10
10 Activity - Results 11
11 Activity - Bias 12
12 Activity - Average estimated SE 13
13 Inertia - Results 14
14 Inertia - Bias 15
15 Inertia - Average estimated SE 16
16 Transitivity - Results 17
17 Transitivity - Bias 18
18 Transitivity - Average estimated SE 19

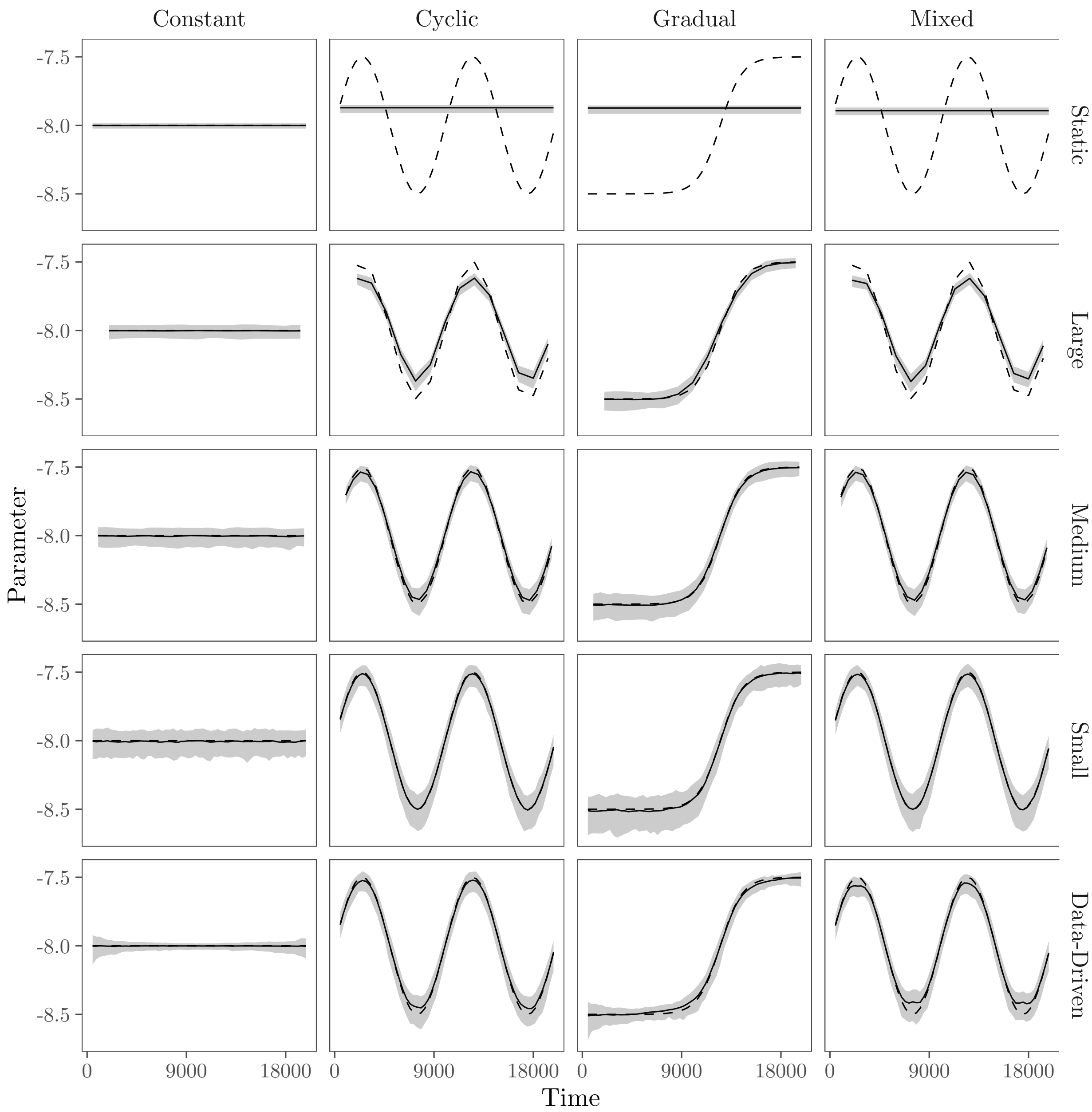

Fig 1. Results from the evaluation of the (moving window) REM for the 'baseline' effect. Rows show results for estimation of the 'baseline' effect with the 'static' REM, large ($4000t$), medium ($2000t$), small ($1000t$), and data-based window widths, respectively. Columns show results for estimation of the 'baseline' effect in the four time-varying effects scenarios. Solid lines represent the mean estimated parameters over 200 datasets over time. The gray area represents the range with 95% of the estimates for the 200 datasets. Dashed lines represent the parameters used for data generation.

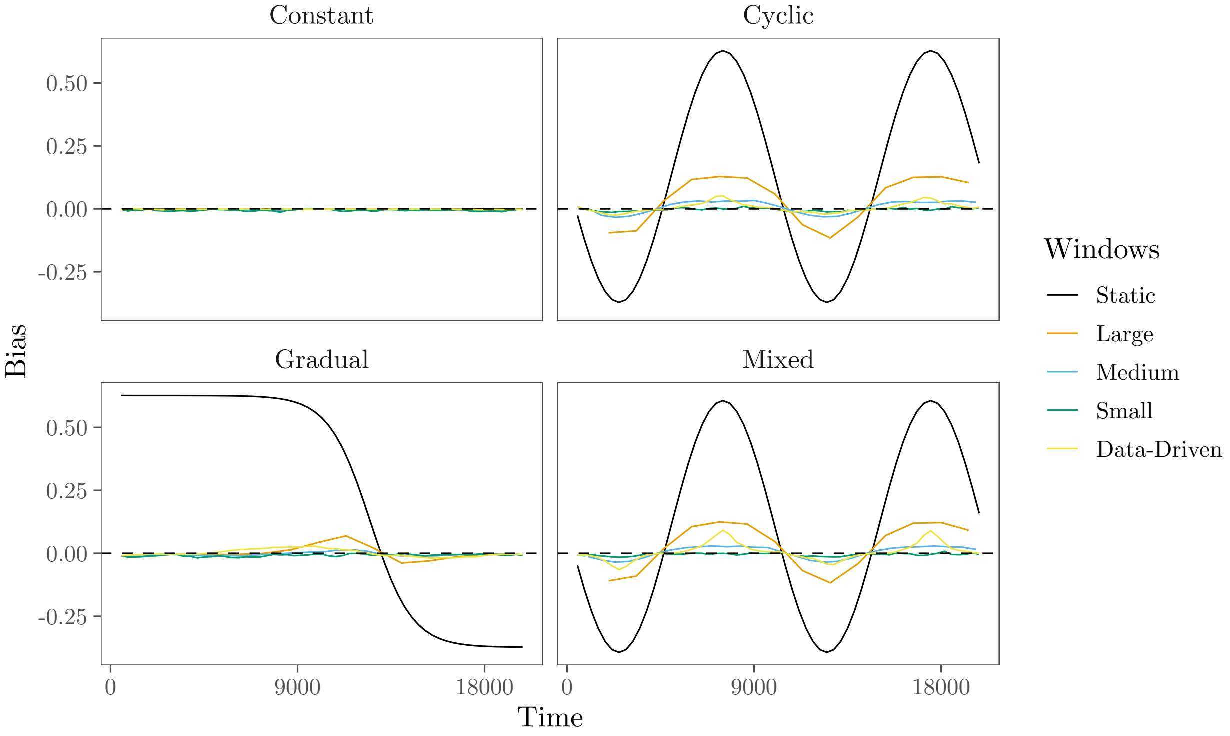

Fig 2. Results for the 'baseline' effect from the evaluation of the moving window REM. Panels refer to the four time-varying effect scenarios. Solid lines represent the bias of the parameter estimates over time, with colors representing estimation with large ($4000t$), medium ($2000t$), small ($1000t$) and data-based window widths.

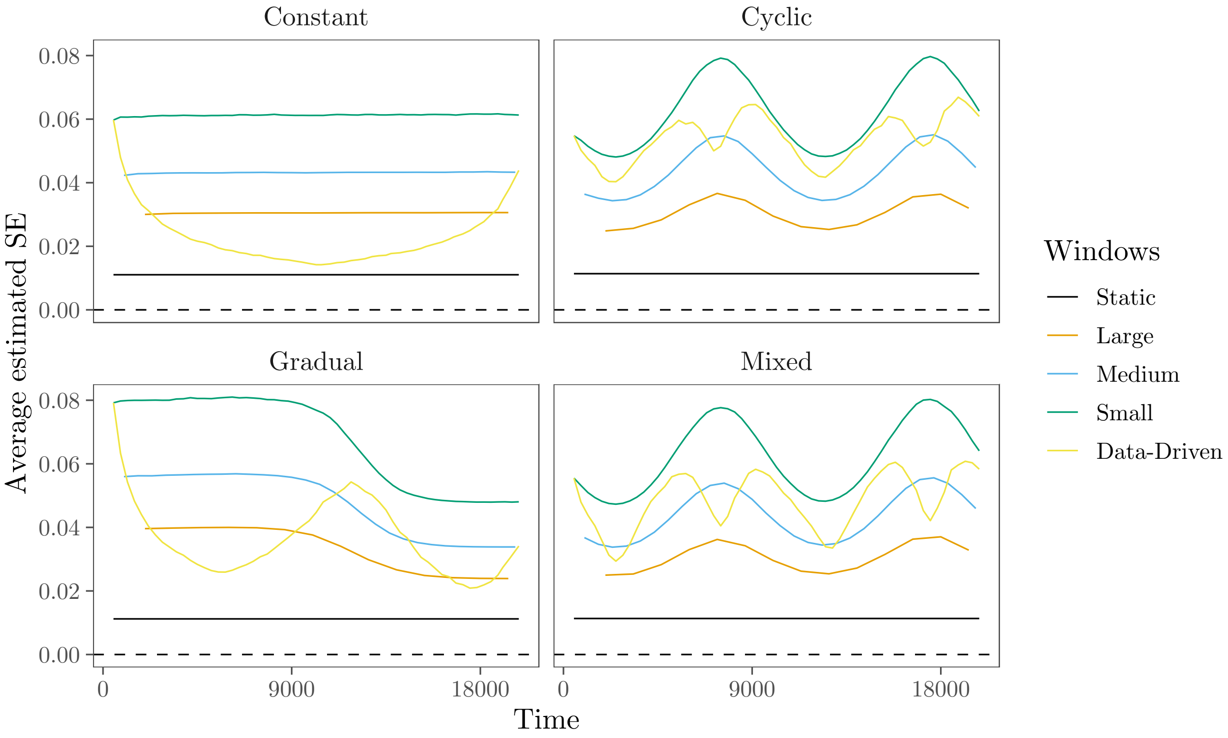

Fig 3. Results for the 'baseline' effect from the evaluation of the moving window REM. Panels refer to the four time-varying effect scenarios. Solid lines represent the average estimated standard error of the parameter estimates over time, with colors representing estimation with large ($4000t$), medium ($2000t$), small ($1000t$) and data-based window widths.

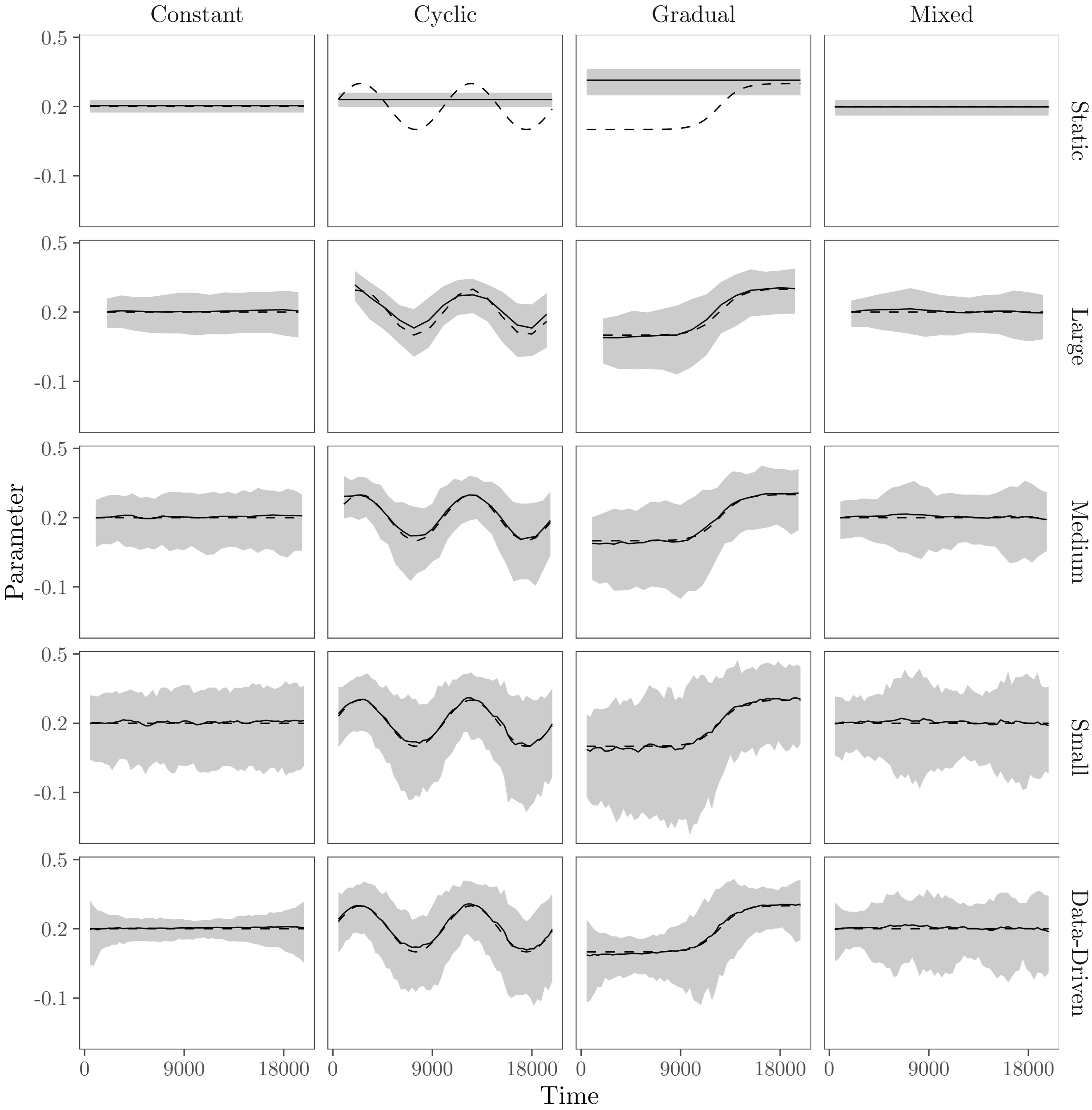

Fig 4. Results from the evaluation of the (moving window) REM for the 'Z of sender' effect. Rows show results for estimation of the 'Z of sender' effect with the 'static' REM, large ($4000t$), medium ($2000t$), small ($1000t$), and data-based window widths, respectively. Columns show results for estimation of the 'Z of sender' effect in the four time-varying effects scenarios. Solid lines represent the mean estimated parameters over 200 datasets over time. The gray area represents the range with 95% of the estimates for the 200 datasets. Dashed lines represent the parameters used for data generation.

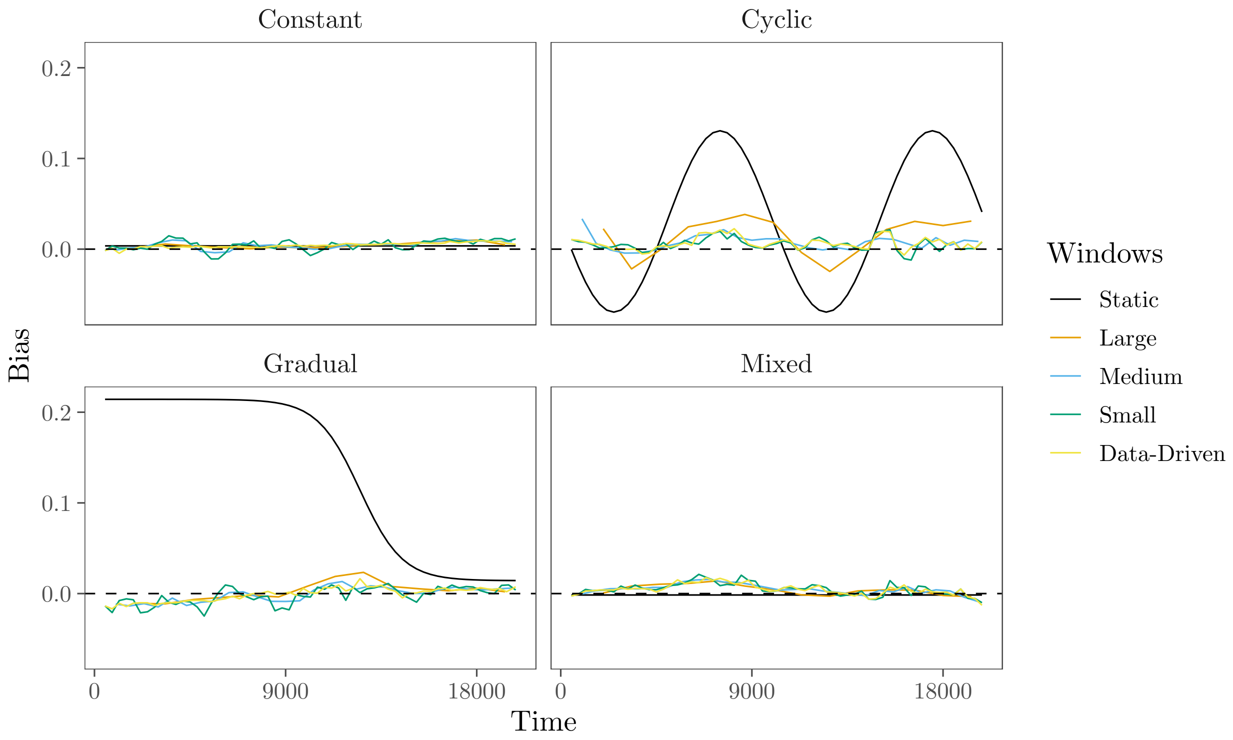

Fig 5. Results for the 'Z of sender' effect from the evaluation of the moving window REM. Panels refer to the four time-varying effect scenarios. Solid lines represent the bias of the parameter estimates over time, with colors representing estimation with large ($4000t$), medium ($2000t$), small ($1000t$) and data-based window widths.

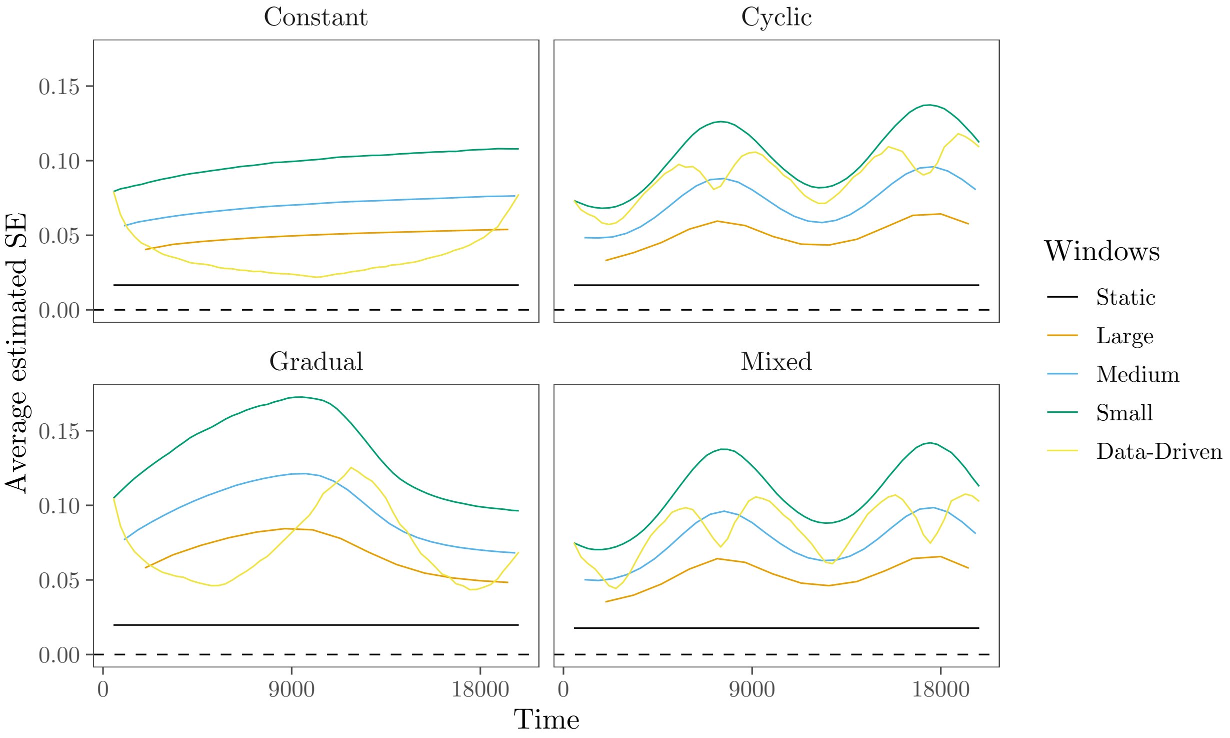

Fig 6. Results for the 'Z of sender' effect from the evaluation of the moving window REM. Panels refer to the four time-varying effect scenarios. Solid lines represent the average estimated standard error of the parameter estimates over time, with colors representing estimation with large ($4000t$), medium ($2000t$), small ($1000t$) and data-based window widths.

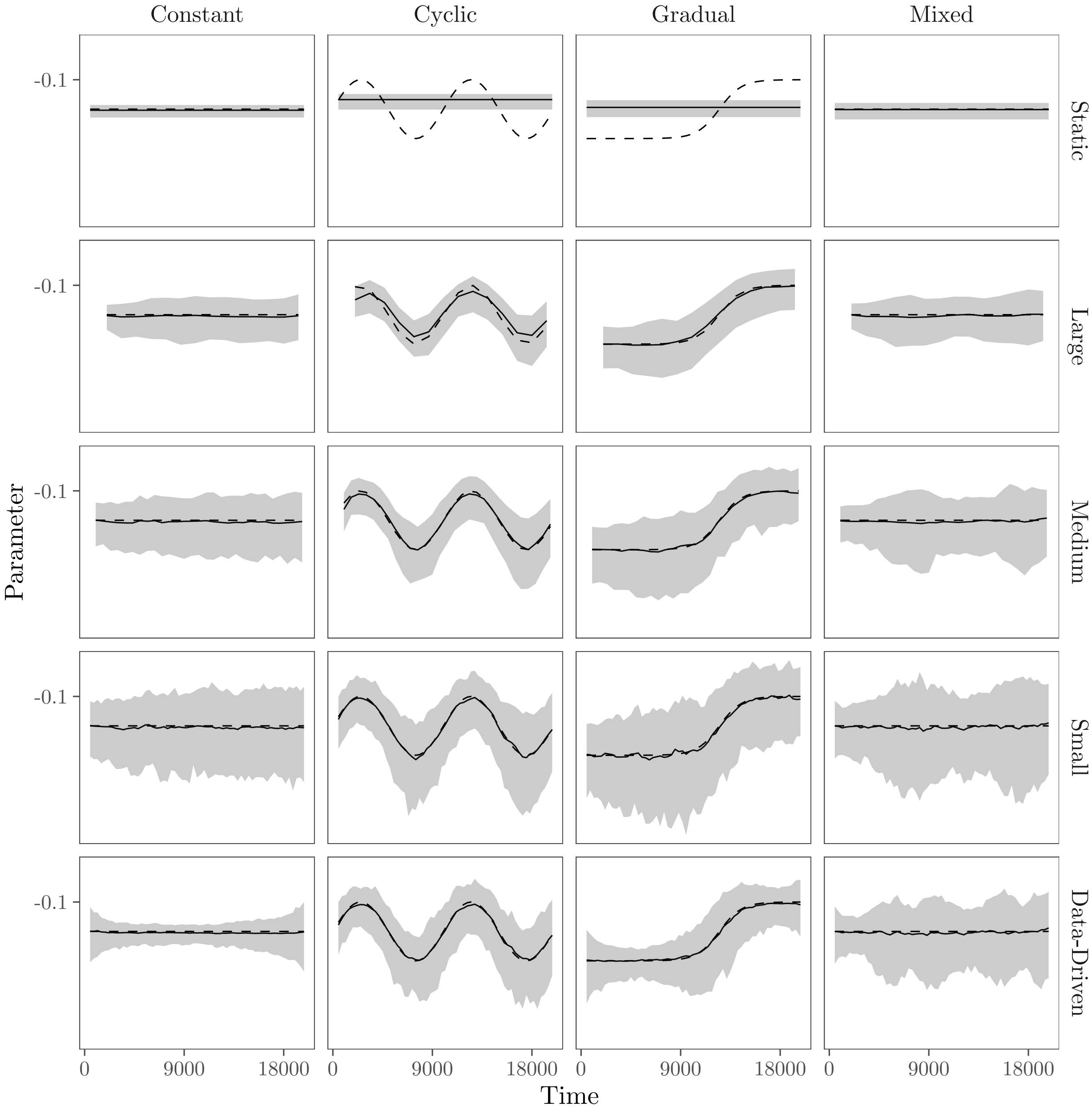

Fig 7. Results from the evaluation of the (moving window) REM for the 'difference in Z' effect. Rows show results for estimation of the 'difference in Z' effect with the 'static' REM, large (4000t), medium (2000t), small (1000t), and data-based window widths, respectively. Columns show results for estimation of the 'difference in Z' effect in the four time-varying effects scenarios. Solid lines represent the mean estimated parameters over 200 datasets over time. The gray area represents the range with 95% of the estimates for the 200 datasets. Dashed lines represent the parameters used for data generation.

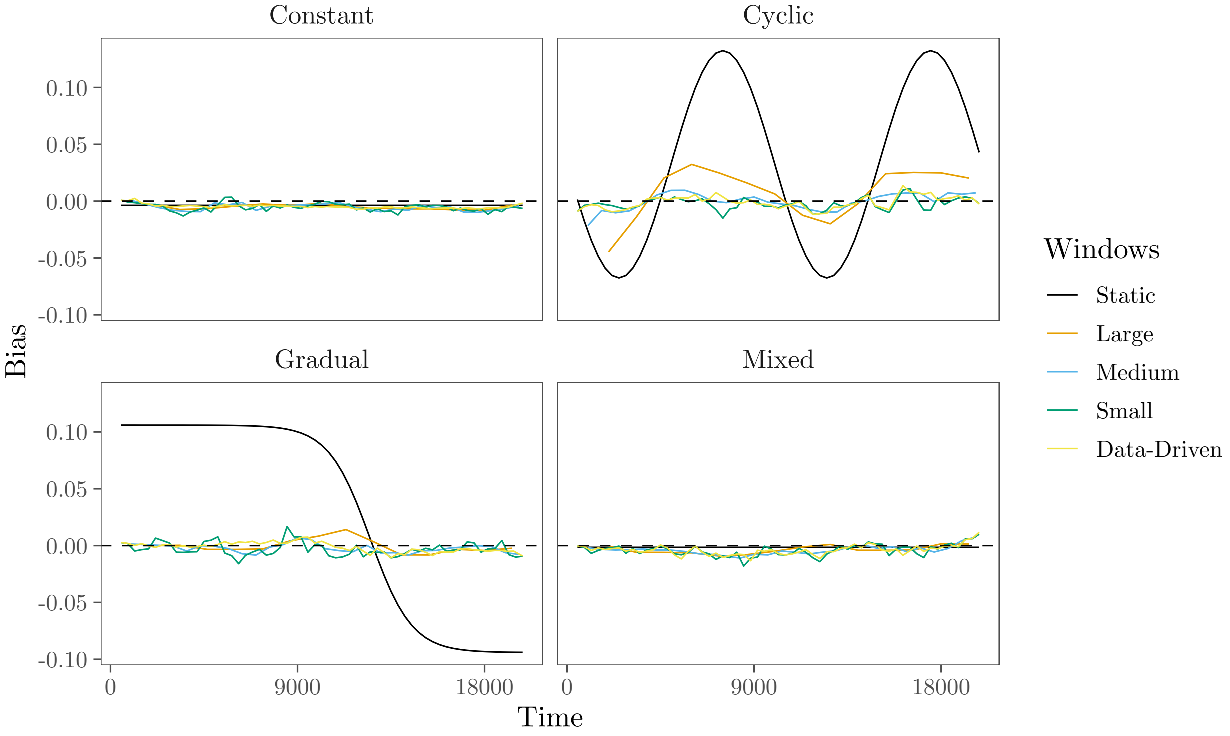

Fig 8. Results for the 'Difference in Z' effect from the evaluation of the moving window REM. Panels refer to the four time-varying effect scenarios. Solid lines represent the bias of the parameter estimates over time, with colors representing estimation with large ($4000t$), medium ($2000t$), small ($1000t$) and data-based window widths.

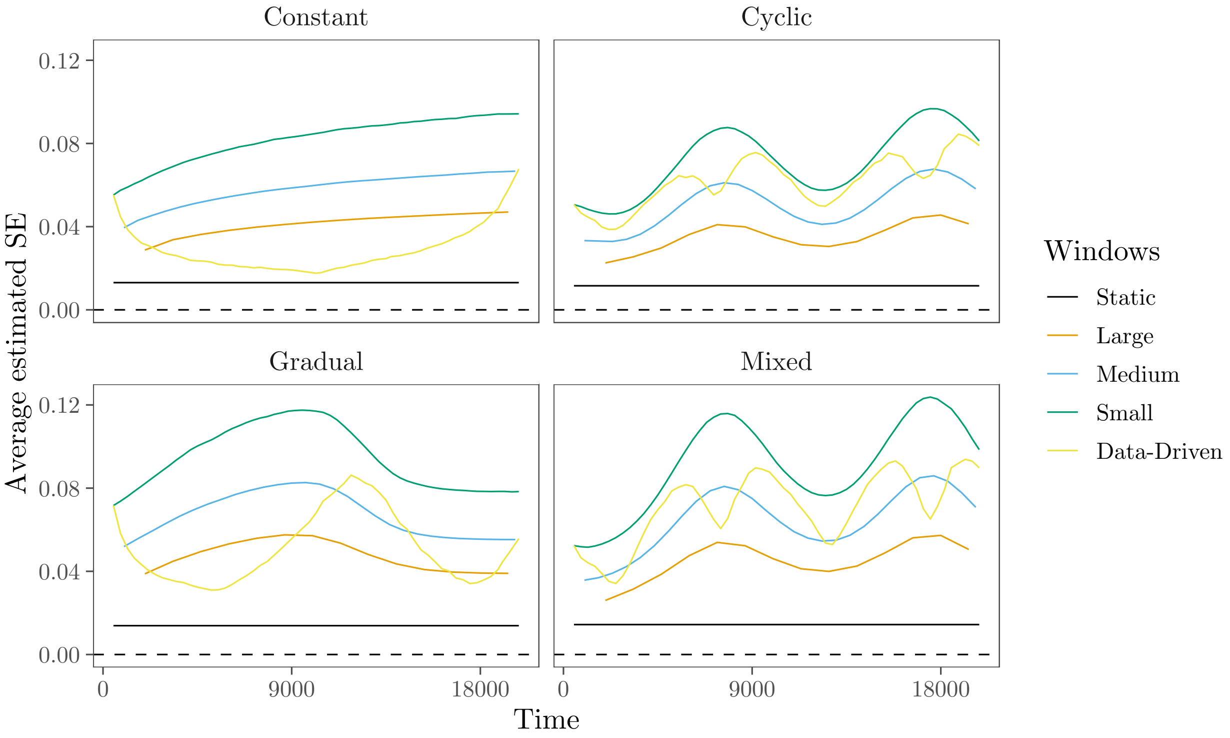

Fig 9. Results for the 'Difference in Z' effect from the evaluation of the moving window REM. Panels refer to the four time-varying effect scenarios. Solid lines represent the average estimated standard error of the parameter estimates over time, with colors representing estimation with large ($4000t$), medium ($2000t$), small ($1000t$) and data-based window widths.

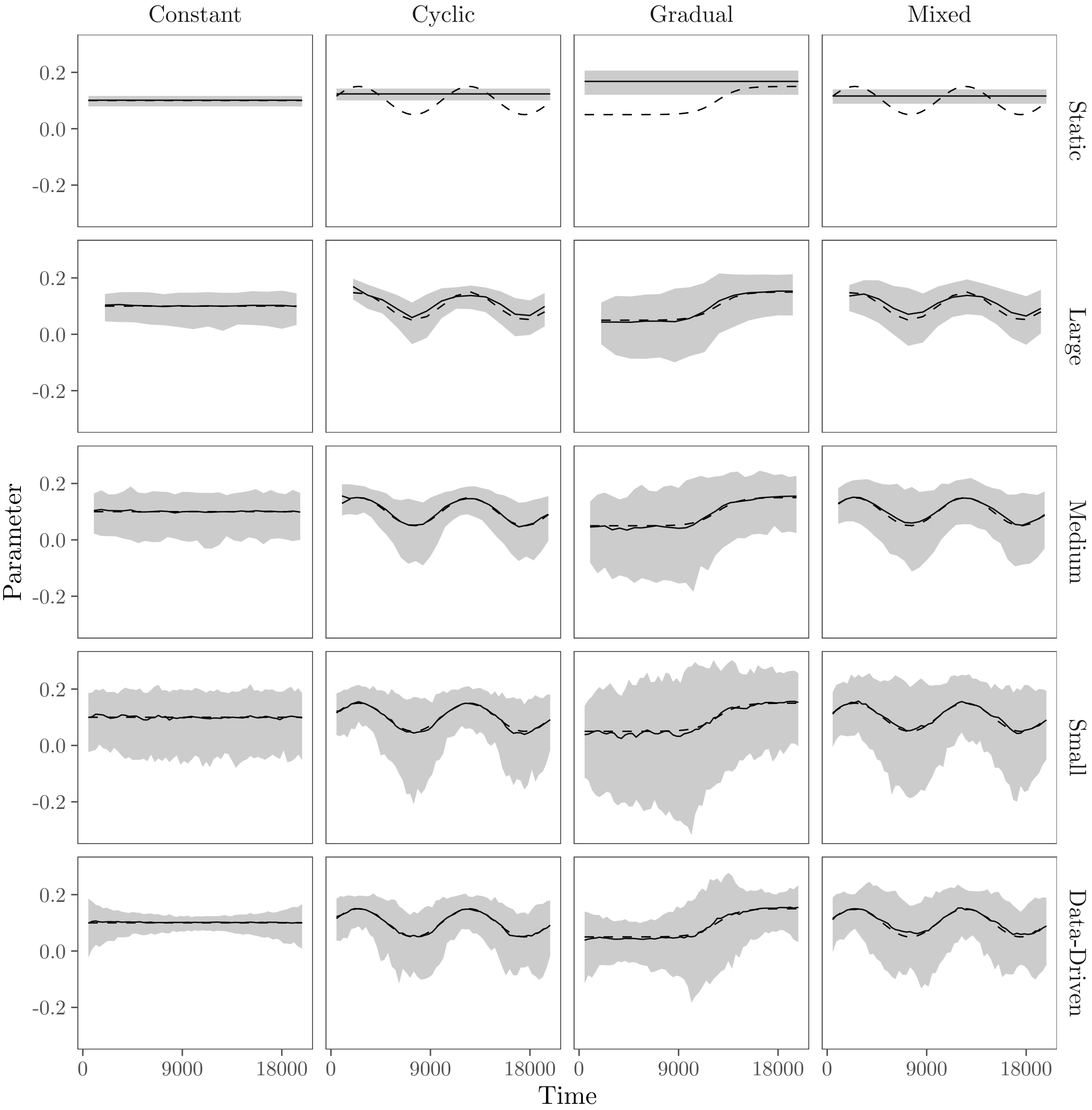

Fig 10. Results from the evaluation of the (moving window) REM for the 'activity' effect. Rows show results for estimation of the 'activity' effect with the 'static' REM, large ($4000t$), medium ($2000t$), small ($1000t$), and data-based window widths, respectively. Columns show results for estimation of the 'activity' effect in the four time-varying effects scenarios. Solid lines represent the mean estimated parameters over 200 datasets over time. The gray area represents the range with 95% of the estimates for the 200 datasets. Dashed lines represent the parameters used for data generation.

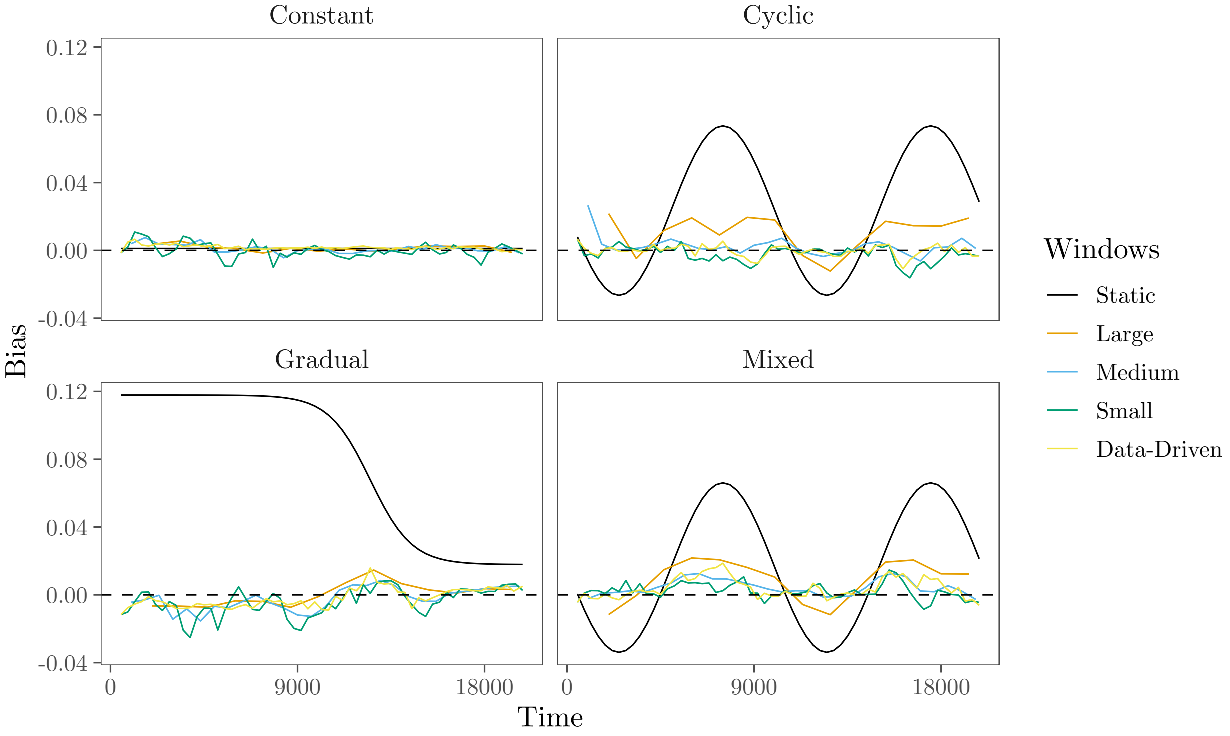

Fig 11. Results for the 'activity' effect from the evaluation of the moving window REM. Panels refer to the four time-varying effect scenarios. Solid lines represent the bias of the parameter estimates over time, with colors representing estimation with large ($4000t$), medium ($2000t$), small ($1000t$) and data-based window widths.

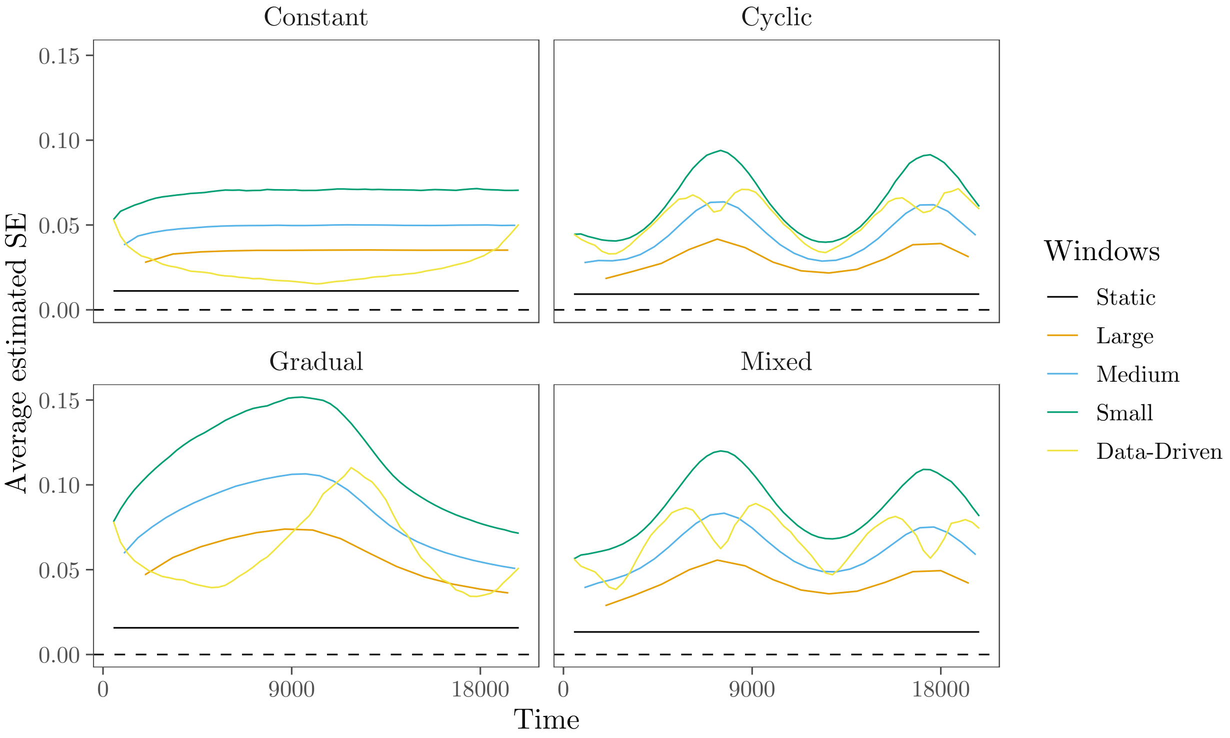

Fig 12. Results for the 'activity' effect from the evaluation of the moving window REM. Panels refer to the four time-varying effect scenarios. Solid lines represent the average estimated standard error of the parameter estimates over time, with colors representing estimation with large ($4000t$), medium ($2000t$), small ($1000t$) and data-based window widths.

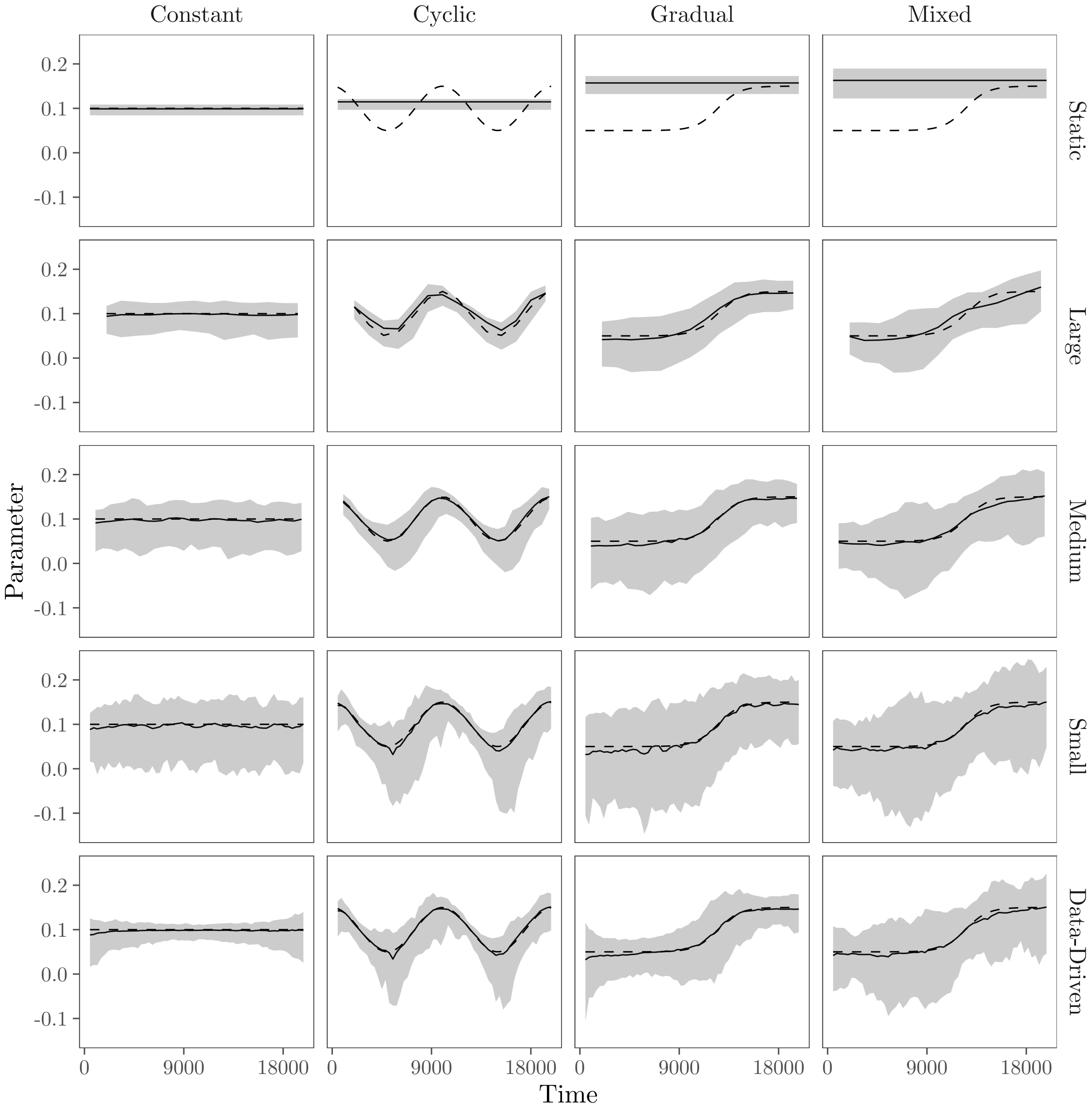

Fig 13. Results from the evaluation of the (moving window) REM for the 'inertia' effect. Rows show results for estimation of the 'inertia' effect with the 'static' REM, large (4000t), medium (2000t), small (1000t), and data-based window widths, respectively. Columns show results for estimation of the 'inertia' effect in the four time-varying effects scenarios. Solid lines represent the mean estimated parameters over 200 datasets over time. The gray area represents the range with 95% of the estimates for the 200 datasets. Dashed lines represent the parameters used for data generation.

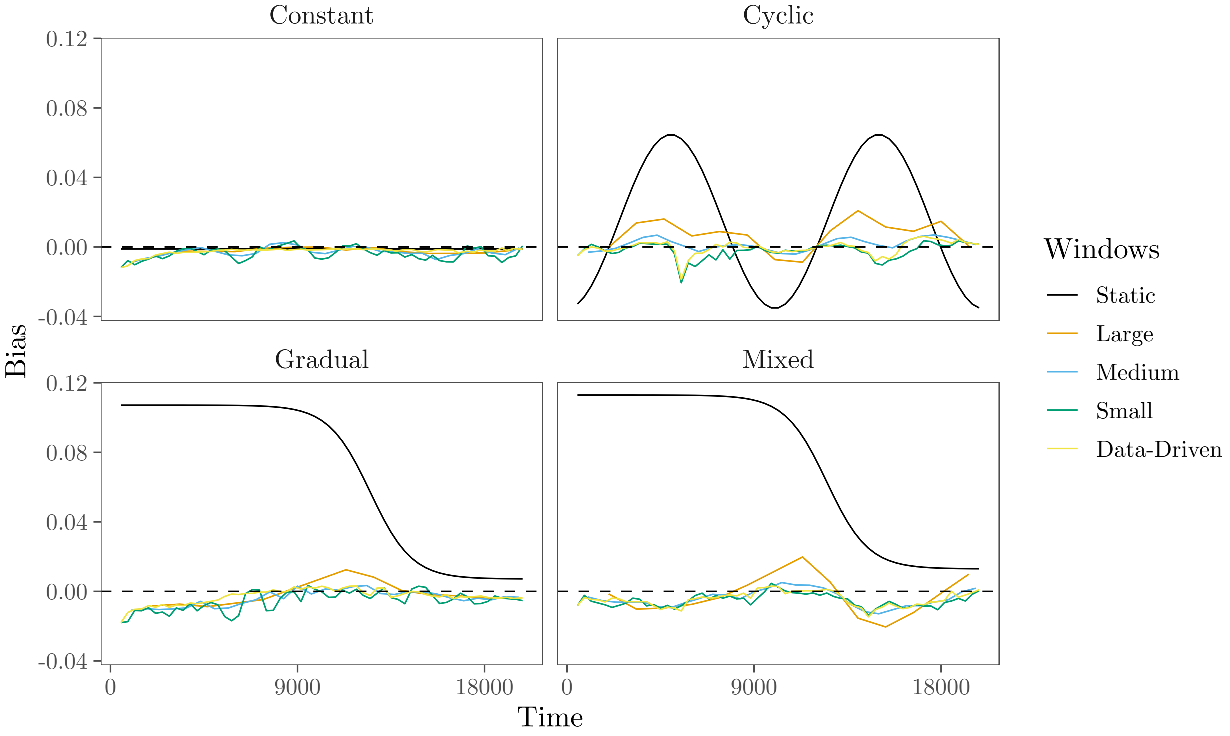

Fig 14. Results for the 'inertia' effect from the evaluation of the moving window REM. Panels refer to the four time-varying effect scenarios. Solid lines represent the bias of the parameter estimates over time, with colors representing estimation with large ($4000t$), medium ($2000t$), small ($1000t$) and data-based window widths.

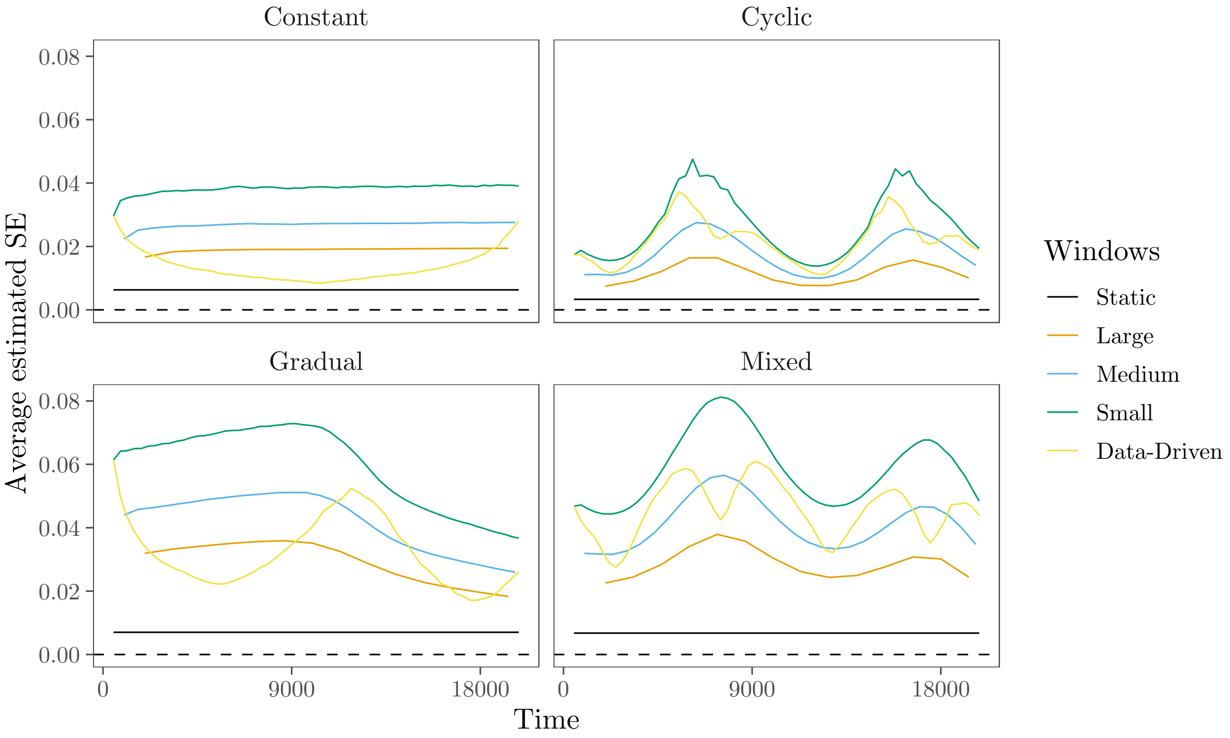

Fig 15. Results for the 'inertia' effect from the evaluation of the moving window REM. Panels refer to the four time-varying effect scenarios. Solid lines represent the average estimated standard error of the parameter estimates over time, with colors representing estimation with large ($4000t$), medium ($2000t$), small ($1000t$) and data-based window widths.

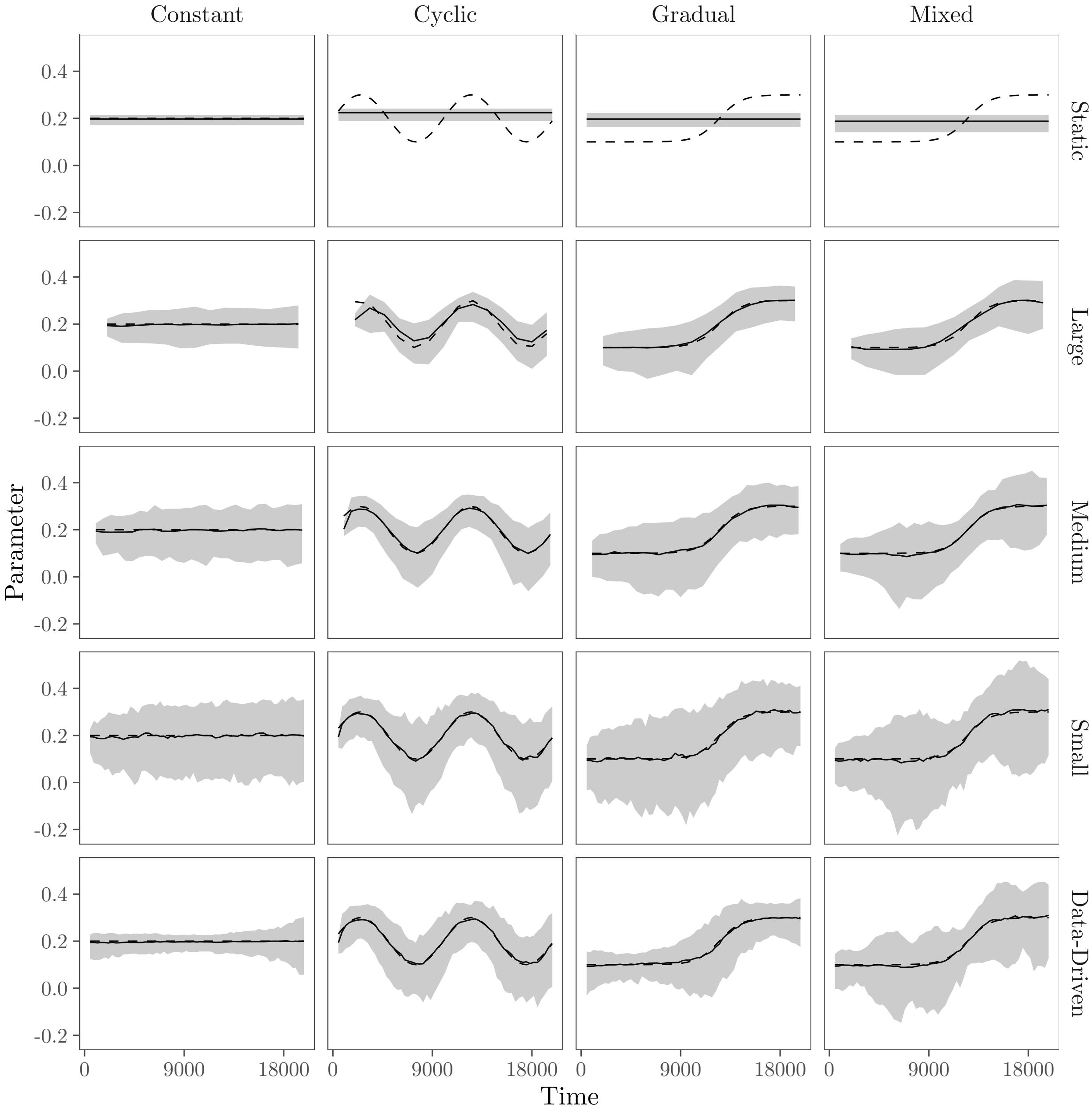

Fig 16. Results from the evaluation of the (moving window) REM for the 'transitivity' effect. Rows show results for estimation of the 'transitivity' effect with the 'static' REM, large ($4000t$), medium ($2000t$), small ($1000t$), and data-based window widths, respectively. Columns show results for estimation of the 'transitivity' effect in the four time-varying effects scenarios. Solid lines represent the mean estimated parameters over 200 datasets over time. The gray area represents the range with 95% of the estimates for the 200 datasets. Dashed lines represent the parameters used for data generation.

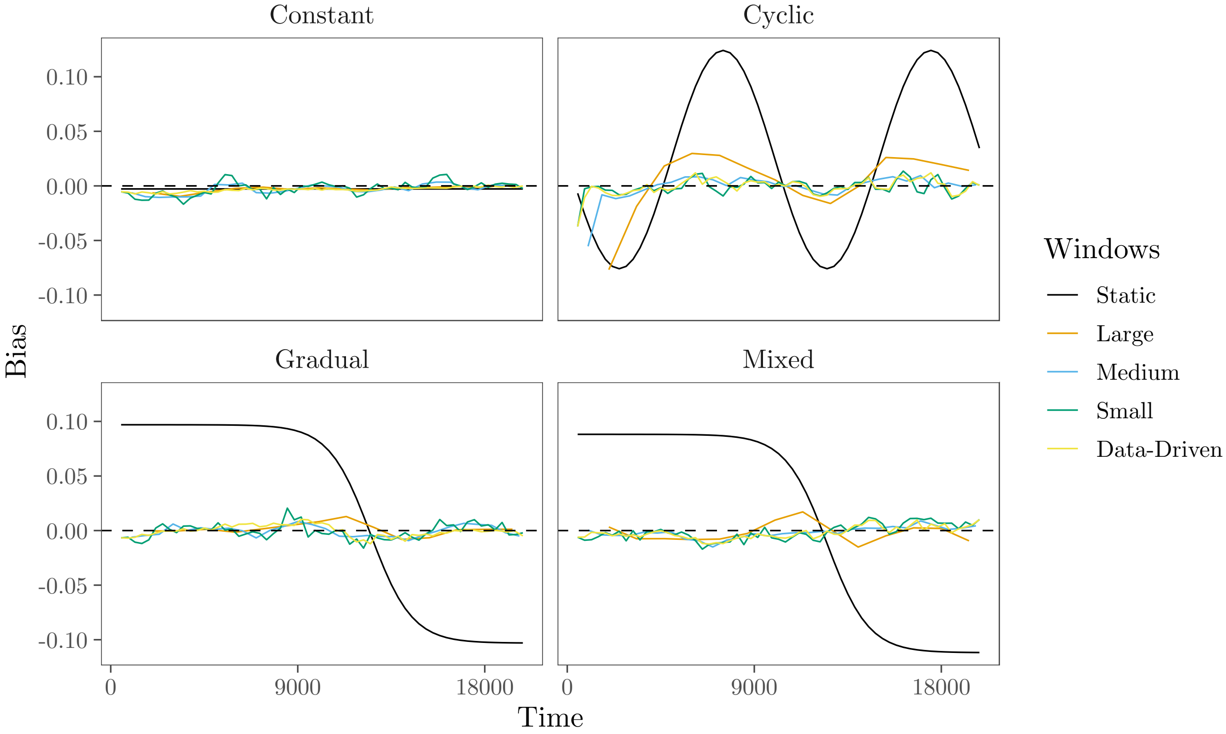

Fig 17. Results for the 'transitivity' effect from the evaluation of the moving window REM. Panels refer to the four time-varying effect scenarios. Solid lines represent the bias of the parameter estimates over time, with colors representing estimation with large ($4000t$), medium ($2000t$), small ($1000t$) and data-based window widths.

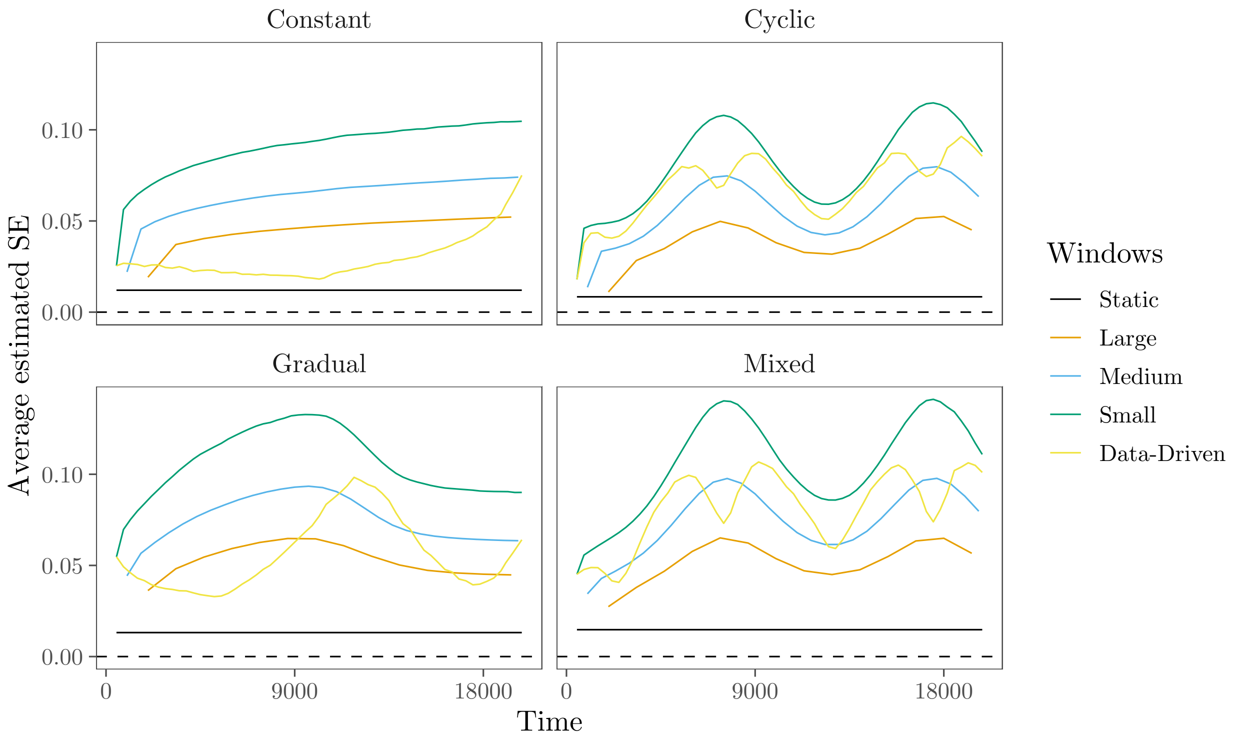

Fig 18. Results for the 'transitivity' effect from the evaluation of the moving window REM. Panels refer to the four time-varying effect scenarios. Solid lines represent the average estimated standard error of the parameter estimates over time, with colors representing estimation with large ($4000t$), medium ($2000t$), small ($1000t$) and data-based window widths.
