# Supplementary material for: Dynamic relational event modeling: Testing, exploring, and applying
Source: PLoS One. 2022 Aug 1;17(8):e0272309. doi: 10.1371/journal.pone.0272309 (PMC9342787; doi:10.1371/journal.pone.0272309)
Supplement: Attachment — Submitted filename: Response to Reviewers.pdf [file pone.0272309.s002.pdf]

Tilburg, June 16, 2022

Dear dr. Shi,

Please find enclosed our revised manuscript “Dynamic relational event modeling: Testing, exploring, and applying”. We want to thank you and the two reviewers for the very positive review of our initial submission.

We took all comments to heart and revised the paper where needed. Below we respond to the specific issues that were raised by the reviewers. Changes that were made to the manuscript are highlighted.

In our manuscript, we describe a dynamic relational event modeling analysis of face-to-face contacts between the employees of an organization. Reviewer 2 suggested that considering additional types of interactions between the employees, e.g., e-mails or online social relationships, may improve our ability to understand the dynamics of the face-to-face contacts. We agree with Reviewer 2 that, when these type of additional data sources are available, considering them in the analysis leads to a richer and more detailed understanding of dynamic social interaction behavior. Unfortunately, we are restricted due to the limited available information in the data. Therefore, we cannot extend the analysis as suggested. We acknowledge this in the Discussion section of the revised manuscript, and state that the proposed model could be extended if such information would be available.

In our Data Availability statement, we previously stated that we will provide repository information for our data at a later stage in the publication process. In the revised manuscript we added the information about the repository with the data and script files to the methods section of our manuscript. The files and instructions necessary to reproduce the results in the manuscript are available at `https://github.com/mlmeijerink/REHdynamics`.

Thank you for considering our manuscript for publication. We look forward to your response.

Sincerely,

Marlyne Meijerink-Bosman
Joris Mulder
Roger Leenders

**Contact information:**
Corresponding author:
Marlyne Meijerink-Bosman
Department of Methodology & Statistics
Tilburg University
P.O. Box 90153
5000 LE Tilburg
The Netherlands
E-mail: m.l.meijerink@tilburguniversity.edu

## Response to reviewer 1

"The paper is a lucid and well-executed methodological article, providing a well-validated method to study temporal parameter heterogeneity in relational event models. It should be of interest to a wide-range of social scientists interested in studying network dynamics. Congratulations on a solid piece of work."

*Response*: Thank you for the positive comments on our manuscript.

## Response to reviewer 2

"This paper extends the REM framework with approaches for testing and exploring time-varying network effects and uses the Bayesian approach and moving window of sliding relationship event history to study time-varying network effects. In addition, the method of this study is applied to an empirical application. This paper finds that estimation with empirically determined window widths achieves both good accuracy for time intervals with important changes and good precision for time intervals with hardly any changes in the effects.

The model in this paper is logical and the results are abundant, interesting and convincing. The conclusions have strong practical significance and reference value. It's a great honor to read this paper, but there are still a few suggestions before publication:"

*Response*: Thank you for the positive comments on our manuscript and suggestions on how we could improve our work. Below we respond to your comments on specific issues.

1. "In the "Recency", should factors other than email be considered? Such as making calls, sending messages, etc."

2. "In addition, whether employees' face-to-face interactions are affected by other factors, such as online social relationships."

*Response to comments 1 and 2*: We agree with you that adding information about additional types of interactions between the employees has the potential to lead to a more rich and detailed understanding of the dynamics of the face-to-face contacts. Unfortunately, we are restricted due to the limited available information in the data. Therefore, we cannot extend the analysis as suggested. We acknowledge this in the Discussion section of the revised manuscript, and state that the proposed model could be extended if such information would be available:

*Moreover, in the current analysis we focus on modeling the predictors of face-to-face interactions between employees in the office space. In many organizations, employees interact via different modes of communication, e.g., face-to-face interactions, e-mail messages, phone calls, etc. In addition, employees may develop social relationships outside of the office space. All these factors are likely to affect the patterns of observed face-to-face interactions between employees in the office space. In the current analysis, we are restricted due to the limited available information in the data. When such information would be available, however, it is recommended to consider it in the configuration of the predictor variables to obtain a more detailed understanding of the dynamics of employees' face-to-face interactions (e.g., see [27]).*

3. “There is a mistake in the use of punctuation in the text, e.g., the sentence on page 25, “have been introduced [e.g.,][]Butts2008,Nooy2011,Perry2013,Stadtfeld2017,Vu2011. Generally, ......”, here I think “have been introduced (e.g., [1,8,22]) Generally, ......” may be correct. Furthermore, Nooy2011 and Stadtfeld2017 were not found in ref.”

*Response*: Thank you for making us aware of these mistakes. We have corrected them in the revised manuscript.

We hope all your comments have been satisfactorily addressed. Thank you again for your review.
